# Associations Among Demographic Factors, Bullying, Victimization and Weight Status Among Children with and Without Current Autism Spectrum Disorder Using the National Survey of Children’s Health

**DOI:** 10.3390/epidemiologia7050121

**Published:** 2026-08-26

**Authors:** Olusegun A. Ogunmola, Laura A. Nabors, Brandon T. Workman, Ashley L. Merianos

**Affiliations:** Health Promotion and Education Program, School of Human Services, University of Cincinnati, Cincinnati, OH 45221, USA

**Keywords:** autism, bullying, school, obesity, children

## Abstract

**Background:** School-aged children belong to a distinct developmental stage in which bullying and weight status can have significant short- and long-term consequences, particularly for those with autism spectrum disorder (ASD). **Methods:** Data were analyzed from the 2023 National Survey of Children’s Health (N = 17,689), including perpetration and victimization among school-aged children. **Results:** They were more likely to be White non-Hispanic compared to multiracial (OR 2.24; 95% CI 2.17–2.31), less likely to be female (OR 0.40; 95% CI 0.38–0.41), younger [6–9 years] (OR 0.42; 95% CI 0.41–0.43), from families within the 100–199% (OR 0.40; 95% CI 0.39–0.42) and 200–299% (OR 0.26; 95% CI 0.26–0.27) poverty level. Adjusted models indicated that children with ASD had higher odds of being underweight (OR 1.75; 95% CI 1.70–1.81) or overweight (OR 3.55; 95% CI 3.37–3.73) than of being obese. They were also more likely to be victimized 1–2 times/week (OR 1.30; 95% CI 1.24–1.37), but less likely to bully others 1–2 times/month (OR 0.31; 95%CI 0.29–0.33). **Conclusions:** These findings suggest that school-aged children with ASD may face elevated risks of victimization and unhealthy weight status.

## 1. Introduction

### Bullying Involvement and Weight Status Among Children with Current Autism Spectrum Disorder Using the National Survey of Children’s Health

The estimated prevalence of autism spectrum disorder (ASD) in the United States (US) for youth aged 3–17 years is about 3% [1,2]. Children with ASD often experience problems with peer interactions and reciprocity in social interactions [3], making them targets of bullying [4]. Researchers investigating factors related to ASD using the 2016 National Survey of Children’s Health (NSCH) found that the odds of victimization are significantly greater for children with ASD than children with other developmental disorders [5]. Dhagustani and colleagues examined parent and teacher reports of victimization and discovered that children with ASD are up to three times more vulnerable to victimization than children who are developing typically [6]. Regarding bullying perpetration, Rowley and colleagues found that children (10–12 years) diagnosed with ASD were more apt to bully others than children who were developing typically [7]. Conversely, other findings suggested that children with ASD were less likely to be perpetrators of bullying, especially when comorbid psychopathology was controlled for [4]. It is important to study bullying and victimization in children with ASD because these factors are related to academic problems, internalizing problems, social isolation, as well as physical and mental health problems in childhood and later years [5,8].

There also may be differences related to sociodemographic factors. Research shows boys are diagnosed with ASD about four times more often than girls [1,9]. Moreover, some research shows that children of racial and ethnic minority groups and those living in households with lower incomes may be more likely to be diagnosed with ASD [9,10,11,12]. Middle childhood (age 6–12 years) is a distinct developmental stage [13,14,15,16]. Bullying and victimization can have substantial social implications during this critical period of socialization since children engage more with their peers during this stage [17,18,19,20].

Children with ASD are at risk of being underweight, overweight or obese compared to typically developing children [21,22]. In Canada, children who have ASD were two times more likely to be underweight than those who do not have ASD even when considering many sociodemographic variables [23]. On the other hand, Sammels and colleagues reviewed studies including both parent reports and data from anthropometric indices and discovered that children who have ASD are 58% more at risk for obesity than children who are developing typically [24]. Kahathuduwa and colleagues indicated that children with ASD in the United States have a 43.7% increased risk of obesity compared to other children [21]. Because results are equivocal, more research is needed about weight status for children with ASD.

The current study assessed bullying and victimization in children with and without ASD using data from the NSCH [25]. Theoretically, this study is grounded in the ecological systems theory [26] and the social–ecological model [27], which posit that health and developmental outcomes are shaped by the interactions between individual attributes and environmental systems/factors. Specifically, this study focuses primarily on child-/individual-level factors (e.g., sex, age, weight status), two critical factors in the mesosystem (bullying and victimization), and poverty, which intersects multiple systems. The current study was a secondary analysis of data from the 2023 NSCH. Our goals were to (1) identify key demographic factors associated with ASD in school-aged children, (2) determine the association between weight status and ASD, (3) determine the association between bullying others and ASD, and (4) examine the association between being bullied by others and ASD. Findings provide information for school and health professionals who work with children with ASD and contribute new knowledge about variables related to ASD within the child and social context, applying social–ecological theories to find associations that will inform interventions for children with ASD.

## 2. Methods

### 2.1. Participants and Procedures

Data for the current study were from the 2023 National Survey of Children’s Health (NSCH). We used the existing dataset and codebook for the NSCH from the Child and Adolescent Health Measurement Initiative [25,28]. The institutional review board at the University of Cincinnati provided non-human subjects approval (2025-0423; date of approval: 16 May 2025) for this study.

The 2023 NSCH was conducted by the Maternal and Child Health Bureau, Health Resources and Services Administration [29]. Households were randomly sampled to identify those with at least one child 0–17 years of age [30]. Parents responded to a survey about one child in the household. Details about the survey (e.g., data content) and data collection are provided by the U. S. Census Bureau [31]. The 2023 NSCH sampled approximately 385,000 households for age-eligible children, and 55,162 surveys were completed [30].

Participants for the current study were 17,801 caregivers/parents providing data on children between the ages of 6 and 12 years. Children with ASD (*n* = 834, 4.7%) and those without ASD (*n* = 16,855, 94.5%) were included in the analyses. We excluded children who did not have a current diagnosis of ASD (*n* = 28, 0.2%) from study analyses. Missing cases (*n* = 84, 0.6%) were excluded from the analyses.

### 2.2. Study Variables

Variables from the 2023 NSCH are found in the 2023 SPSS Codebook for the NSCH [28]. The dependent variable of interest was parent report of whether they were told their child currently had autism or ASD (Autism_23; referred to as ASD in this manuscript). Parents were asked, “Has a doctor or health care provider ever told you that this child has autism or autism spectrum disorder?” Responses coded “yes” (coded as 1) or “no” (coded as 0) were included in the analysis. Predictors were age, biological sex, race/ethnicity of the child, family income level, BMI of the child, bullying others, and being bullied. Sex of child (Sex_23) was coded “male” (code 0) and “female” (code 1). Parents were asked to identify their child’s race and ethnicity (Race4_23). We considered “Hispanic” (code 0), “White, non-Hispanic” (code 1), “Black, non-Hispanic” (code 2), and “multiracial, non-Hispanic” (code 3). Information on family income was based on the imputed family poverty level variable (Povlev4_23). Four response categories were considered: “0–99% of poverty level PL” (code 0), “100–199% of PL” (code 1), “200–399% of PL” (code 2), and “400% or more of PL” (code 3). Four response categories were considered for BMI status (BMI4_6to17_23): “underweight” (lower than the 5th percentile; code 0), “normal weight” (5th–84th percentile; code 1), “overweight” (85th–94th percentile; code 2), and “obese” (greater than or equal to the 95th percentile; code 3). Bullying others (bully_23) was measured with the question, “During the past 12 months, how often did this child bully others, pick on them, or exclude them, age 6–17 years?” Responses were coded as follows: “never in past 12 months” (code 0), “1–2 times in the past 12 months” (code 1), “1–2 times per month” (code 2), “1–2 times per week” (code 3), and “almost every day” (code 4). Being bullied (bullied_23) was measured with the question, “During the past 12 months, how often was this child bullied, picked on, or excluded by other children, age 6–17 years?” Five responses were considered for analyses: “never in the past 12 months” (code 0), “1–2 times in the past 12 months” (code 1), “1–2 times per month” (code 2), “1–2 times per week” (code 3), and “almost every day” (code 4). Age was categorized as: children (ages six to nine years, code 0) and pre-adolescents (ages 10 to 12 years, code 1). Research has supported adolescence as beginning at age ten [32] and has established ages 10–12 years as pre-adolescent or preteen (“Tween”) years [33]. Research also supports the notion that pre-adolescents aged 10–12 years are a unique social group [34].

### 2.3. Data Analysis

Data were obtained from the 2023 dataset for the NSCH [24]. Raw counts and weighted frequencies and proportions, adjusted using the survey sampling weight (FWC), were reported for the child-level predictors. The weighting variable (FWC) is adjusted for non-response rate and is described in the technical report for the NSCH [31]. A weighted multinomial logistic regression model was used to compare children with and without ASD for the predictors, which included sex of the child, race, poverty level, BMI classification, bullying others, being bullied by others, and age. The model was weighted using the FWC variable. All data analyses were performed using IBM SPSS software version 29 [35].

## 3. Results

There were 17,689 children (6–12 years) in our sample. Table 1 presents demographic information of the participants and results of chi-square tests to examine differences between children with ASD and without ASD. Eight hundred thirty-four parents (4.7%) reported that they had been told that their child had ASD. A total of 16,855 (95.3%) children did not have ASD. Children with ASD were mostly male (73.7%) and White, non-Hispanic (44.6%). Children with ASD were from lower-income families (0–99% poverty level; 24.8%) compared to children without ASD (16.8%). Additionally, those with ASD had higher BMI levels (overweight; 12.3% vs. 4.4%), engaged in lower levels of bullying others (never in the past 12 months; 74.2% vs. 70.5%), and were bullied by others more frequently than children without ASD (1–2 times in the past 12 months; 28% vs. 17.9%) (see Table 1).

The results of the weighted multinomial logistic regression, including odds ratios and confidence intervals, are presented in Table 2. Children with ASD were compared to those without ASD (referent group). The chi-square for the model was significant (*p* < 0.001). Age and biological sex were significant predictors of ASD. Children aged 6–9 years were 58% less likely to have ASD (OR = 0.42; 95% CI, 0.41–0.43, *p* < 0.001) compared to those aged 10–12 years. Female children were 60% less likely to have ASD (OR = 0.40; 95% CI, 0.38–0.41, *p* < 0.001) compared to male children. Race was a significant predictor of ASD. Children who were Hispanic (OR = 1.58; 95% CI, 1.53–1.63, *p* < 0.001), White, non-Hispanic (OR = 2.24; 95% CI, 2.17–2.31, *p* < 0.001), and Black, non-Hispanic (OR = 2.26; 95% CI, 2.18–2.34, *p* < 0.001), were more likely to have ASD compared to children who were multiracial. The strongest associations were between White, non-Hispanic and Black children (approximately 2.2 times greater odds of ASD). Those residing in the lowest-income group (0–99% poverty level) had about two times greater odds of ASD (OR = 1.86; 95% CI, 1.77–1.95, *p* < 0.001) compared to those in the highest-income group. Further, those residing at the 100–199% poverty level (OR = 0.40; 95% CI, 0.39–0.42, *p* < 0.001) and 200–399% (OR = 0.26; 95% CI, 0.25–0.27, *p* < 0.001) poverty level were less likely to have ASD compared to those at the highest-income level (400% or more).

Children with ASD were more likely to be underweight (OR = 1.75; 95% CI, 1.70–1.81, *p* < 0.001), of normal weight (OR = 1.44; 95% CI, 1.41–1.48, *p* < 0.001), and overweight (OR = 3.55; 95% CI, 3.37–3.73, *p* < 0.001) than obese. Children with ASD were more likely to never bully in the past 12 months (OR = 1.18; 95% CI, 1.10–1.27, *p* < 0.001) compared to bullying almost every day. Children with ASD were less likely to bully others one to two times in the past 12 months (OR = 0.66; 95% CI, 0.61–0.71, *p* < 0.001) or one to two times per month (OR = 0.31; 95% CI, 0.29–0.33, *p* < 0.001) compared to bullying almost every day.

Lastly, being bullied by others was a significant predictor of ASD. Children with ASD were less likely to never be bullied in the past 12 months (OR = 0.43; 95% CI, 0.41–0.45, *p* < 0.001) and one to two times in the past 12 months (OR = 0.87; 95% CI, 0.84–0.91, *p* < 0.001) compared to being bullied almost every day. Children with ASD were more likely to be bullied one to two times per month (OR = 1.77; 95% CI, 1.67–1.89, *p* < 0.001) or 1–2 times per week (OR = 1.30; 95% CI, 1.24–1.37, *p* < 0.001) compared to being bullied almost every day (Table 2).

## 4. Discussion

This study examined the association among demographic factors, bullying, victimization, and weight status in children with ASD and their peers who were developing typically. Consistent with the literature, children with ASD were more likely to be male and older, ten to twelve years of age [1,36]. Study findings also indicated that children with ASD had lower odds of bullying others weekly or monthly (compared to daily) when compared to their peers who are developing typically. Moreover, children with ASD were more likely to be bullied by others, which unfortunately is consistent with the literature [5] and concerning when one considers the negative impact of being bullied on child mental health and social status [37]. Compared to the obese category, underweight, normal weight and overweight categories were higher in children with ASD, with overweight being the most common (3.55 times greater odds). Our results are consistent with the idea that children with ASD may be underweight [23], and health professionals need to be aware of this and provide guidance for healthy eating and referral for nutrition counseling as needed. On the other hand, previous research suggests that children with ASD are likely to be obese [24]. However, we found that it is more likely they were overweight than obese, which is encouraging. Nonetheless, parents may benefit from receiving guidance for planning healthy meals and reducing calories in meals for children with ASD who are overweight [38].

Youth who were White, Black, or Hispanic were more likely to report an ASD diagnosis compared to children who were multiracial. This is consistent with a study based on the 2016–2018 cohort of the NSCH, where among children in middle childhood (6–11 years), the prevalence of ASD was lower among the other/multiracial group compared to the other races [36]. In contrast, Salehi and colleagues found a high prevalence of ASD among multiracial children [1]. Further exploration of variables that might influence differences in diagnosis across racial/ethnic groups is needed. Some variables to consider are health care access, cultural influences on service utilization, and inconsistencies in diagnostic criteria [39,40].

Results demonstrated that parents of boys were about three times more likely to state they had been told their child had ASD than if the child was a girl. Other literature supports this finding [1,9]. As mentioned, age was significantly associated with a current diagnosis of ASD, with parents of older children (ages 10–12) having higher odds of being told their child had ASD than parents of younger children. This finding is consistent with a similar study based on the NSCH among 3–17-year-olds, where it was found that the prevalence of ASD increased with age [1]. Perhaps the increased diagnostic stability of ASD with age and the increase in screening opportunities within school settings are related to the age difference [41]. Unfortunately, younger children may receive more school-based services [42]. It will be important to examine services provided for older youth (preteens and teens) in future studies and how this contributes to ASD identification, especially in comparison to school-based services for younger children.

Results for poverty level varied. Parents of children from families at the lowest poverty level (0–99%) reported their child had ASD more often than those at the higher poverty level (≥400%), whereas parents of children from families at the “middle” (100–199% and 200–399%) poverty levels were less likely to reporte their childas having ASD diagnosis compared to those at the highest poverty (≥400%) level. A recent study based on the 2020–2021 NSCH found a significantly higher prevalence of ASD among children whose families were below the 100% poverty level [1]. Moreover, experts from the Autism and Developmental Disorders Network (ADDN) have noted that ASD is now more commonly diagnosed in children from low-income families, whereas the diagnosis used to be more commonly associated with high-income families [9]. However, the highest-income group had higher levels than those in the middle-level income groups, suggesting a non-linear relationship between family income and odds of reported ASD diagnosis. This could be due to residual confounding with another variable, such as access to medical care. Future studies should examine factors related to ASD in different income-level groups to uncover reasons parents of youth in middle-income families may be less likely to be told their child has ASD, which can serve as a basis for tailoring interventions.

Compared to their typically developing peers, children with ASD were more likely to be underweight, normal weight, or overweight, compared to being obese. However, the odds of being overweight compared to obese were higher than the other two weight categories. Results of previous studies have indicated a higher risk of overweight/obesity among children with ASD, suggesting a need for improved healthy eating and exercise for this vulnerable group [21,24,43]. On the other hand, children who have ASD were also more likely to be underweight, which could be due to selective eating [44]. Selective eating can limit the variety in one’s diet, resulting in inadequate nutrient intake and increased risk of being underweight [44,45,46]. This idea is speculative. Future studies should explore the role of eating behaviors related to food selectivity and being underweight. Understanding more about relations among eating patterns, physical activity, and weight status among children with ASD may provide useful information for developing interventions [47].

Children who were more likely to be bullied had higher odds of having ASD. Research using an earlier version of the NSCH also showed that children who have ASD are more likely to be bullied than children who do not have ASD [5]. Similarly, Käld and colleagues indicated that children with ASD faced bullying [48]. This is concerning due to the negative mental health impact of victimization [5,8]. Understanding reasons for bullying and designing interventions to reduce peer bullying are needed. Social impairments in children with ASD may be related to lower levels of peer interaction and possibilities for being bullied, and subsequently hinder the development of friendships [7,48]. It will remain important to foster self-esteem and peer relationships for children with ASD [37]. Moreover, further research is needed to test interventions to support positive peer interactions of elementary school-age children and preteens who have ASD with their peers [49] and then assess whether victimization rates decrease for children with ASD.

## 5. Limitations

Several things may have influenced study findings. First, this study was cross-sectional, and as such, it does not address changes over time in bullying and victimization, which is a critical area for study. Second, this sample had more White children and children who lived in families with higher income levels, and more information about relations among demographic factors, bullying, victimization, and weight status will inform health professionals working with children of color who reside in low-income families. Also, the NSCH relies on self-report from parents, and they may have misunderstood diagnostic information about ASD status and child weight provided by health professionals. Information about diagnoses provided by health professionals may provide more accurate information about whether a child meets criteria for ASD or is over- or underweight. Similarly, parent report of bullying/victimization could have been incorrect, because they did not know about these experiences or their reports were biased, in that they under-reported this information. Teacher perceptions or behavioral observations may provide more information on variables related to bullying/victimization of children with ASD.

## 6. Conclusions

Parent report indicated older children, ages ten to twelve, were more likely to be identified as having ASD, and providing services for this age group should continue to be a priority. Findings suggested that victimization may be higher in children with ASD compared to those without ASD. Increasing knowledge of health professionals and educators about this fact and improving their knowledge of interventions to improve peer interactions and support children with ASD who have been bullied will enhance the social development of children in this vulnerable group. While it is encouraging that children with ASD were more likely to have normal weight (relative to being obese) when compared to children without ASD, our findings also showed that school-aged children with ASD were more likely to be reported as underweight and overweight compared to those without ASD. Improved awareness, screening, and support regarding nutrition and physical activity for children with ASD may be necessary to improve weight-related health outcomes. In the future, longitudinal studies, using interviews, observations, and surveys with multiple informants, may yield more detailed information about interrelations between family income levels, race group, weight status, and social interactions of children with ASD to inform interventions for this vulnerable group.

## Figures and Tables

**Table 1:** Demographic information for children diagnosed with ASD and those without ASD.

		ASD Status ^a^		
Variable		Yes (*n* = 834)	No (*n* = 16,855)	*p*-Value
		*n* (%) ^b^	*n* (%)	
Age Groups				*p* < 0.001
	6–9 years	486 (57.3)	9640 (73.7)	
	10–12 years	348 (42.7)	7215 (26.3)	
Sex				*p* < 0.001
	Male	633 (73.4)	8580 (90.3)	
	Female	201 (26.6)	8275 (9.7)	
Race				*p* < 0.001
	Hispanic	118 (20.9)	2476 (37.8)	
	White, non-Hispanic	510 (44.6)	10,827 (32.5)	
	Black, non-Hispanic	82 (23.6)	1034 (14.2)	
	Multiracial, non-Hispanic	124 (11)	2518 (15.4)	
Poverty Level				*p* < 0.001
	0–99%	153 (24.8)	2156 (16.8)	
	100–199%	179 (21.7)	2712 (23.6)	
	200–399%	249 (32.3)	5056 (49.8)	
	400% or more	253 (21.2)	6931 (9.8)	
Weight Status ^c^				*p* < 0.001
	Underweight	90 (11.7)	1758 (17.6)	
	Normal	406 (53.6)	9222 (52.1)	
	Overweight	120 (12.3)	2361 (4.4)	
	Obese	162 (22.3)	2471 (26)	
Bully Others (Past 12-Months)				*p* < 0.001
	Never in the past 12 months	590 (74.2)	13,219 (70.5)	
	1–2 times in the past 12 months	139 (14.8)	2823 (11.7)	
	1–2 times per month	48 (4.9)	398 (8.7)	
	1–2 times per week	38 (4.5)	151 (6.7)	
	Almost every day	12 (1.7)	54 (2.5)	
Bullied by Others (Past 12-Months)				*p* < 0.001
	Never in the past 12 months	261 (40.8)	8529 (57.7)	
	1–2 times in the past 12 months	238 (28)	5992 (17.9)	
	1–2 times per month	124 (9.1)	1478 (4.6)	
	1–2 times per week	121 (13.3)	635 (11.6)	
	Almost every day	82 (8.7)	278 (8.3)	

^a^ N = 17,689; ^b^ The information in this table represents raw counts, weighted percentages, and *p*-values from weighted chi-square tests; ^c^ Underweight was defined as less than 5th percentile, normal weight as 5th–84th percentile, overweight as 85th–94th percentile, and obese as 95th percentile and higher.

**Table 2:** Weighted multinomial logistic regression results for sociodemographic variables, weight status, bullying, and victimization for ASD status ^a^.

Variable		Odds Ratio	Confidence Interval		*p*-Value
			Lower	Upper	
Age					
	6–9 years	0.42	0.41	0.43	<0.001
	10–12 years (referent group)				
Sex					
	Female	0.40	0.38	0.41	<0.001
	Male (referent group)				
Race					
	Hispanic	1.58	1.53	1.63	<0.001
	White, non-Hispanic	2.24	2.17	2.31	<0.001
	Black, non-Hispanic	2.26	2.18	2.34	<0.001
	Multiracial, non-Hispanic (referent group)				
Poverty Level					
	0–99%	1.86	1.77	1.95	<0.001
	100–199%	0.40	0.39	0.42	<0.001
	200–399%	0.26	0.26	0.27	<0.001
	400% or more (referent group)				
Weight Status					
	Underweight	1.75	1.70	1.81	<0.001
	Normal	1.44	1.41	1.48	<0.001
	Overweight	3.55	3.37	3.73	<0.001
	Obese (referent group)				
Bully Others (Past 12-Months)					
	Never in the past 12 months	1.18	1.10	1.27	<0.001
	1–2 times in the past 12 months	0.66	0.61	0.71	<0.001
	1–2 times per month	0.31	0.29	0.33	<0.001
	1–2 times per week	1.11	0.93	1.11	0.749
	Almost every day (referent group)				
Bullied by Others (Past 12-Months)					
	Never in the past 12 months	0.43	0.41	0.45	<0.001
	1–2 times in the past 12 months	0.87	0.84	0.91	<0.001
	1–2 times per month	1.77	1.67	1.88	<0.001
	1–2 times per week	1.30	1.24	1.37	<0.001
	Almost every day (referent group)				

^a^ Reference group for the predictor was ‘no history of ASD’.

## Data Availability

The 2023 NSCH was used, a public dataset available on request from the Data Resource Center for Child and Adolescent Health at https://www.childhealthdata.org/help/dataset (accessed on 11 May 2025).
